# Supplementary material for: Sphinganine recruits TLR4 adaptors in macrophages and promotes inflammation in murine models of sepsis and melanoma
Source: Nat Commun. 2024 Jul 18;15:6067. doi: 10.1038/s41467-024-50341-w (PMC11258287; doi:10.1038/s41467-024-50341-w)
Supplement: Supplementary file 1 — Supplementary Information [file 41467_2024_50341_MOESM1_ESM.pdf]

SUPPLEMENTARY INFORMATION

Supplementary Fig. 1, related to Figure 1 and Figure 3. Sptlc2 levels are diminished in BMDM of *Lyz2*-cre mice.

Supplementary Fig. 2, related to Figure 2. *Sptlc2* deficiency impairs M1-like BMDM growth.

Supplementary Fig. 3, related to Figure 3. *Sptlc2* deficiency inhibits M1-like macrophage growth by preventing MyD88 recruitment to TLR4, which cannot be entirely rescued through overexpression of WT or L252P-mutated MyD88.

Supplementary Fig. 4, related to Figure 4. Sphinganine physically interacts with TIRAP and MyD88.

Supplementary Fig. 5, related to Figure 1 and Figure 5. *Sptlc2*-deficient mice, showing attenuated LPS-induced sepsis symptoms and plasma cytokine levels.

Supplementary Fig. 6, Deficiency in *Sptlc2* alleviates pro-inflammatory M1-like macrophage phenotype and cytokine production in cecal slurry-induced polymicrobial sepsis

Supplementary Fig. 7, related to Figure 6. Myeloid cell-specific Sptlc2 shapes the anti-tumor immune response in the B16-F10 melanoma mouse model.

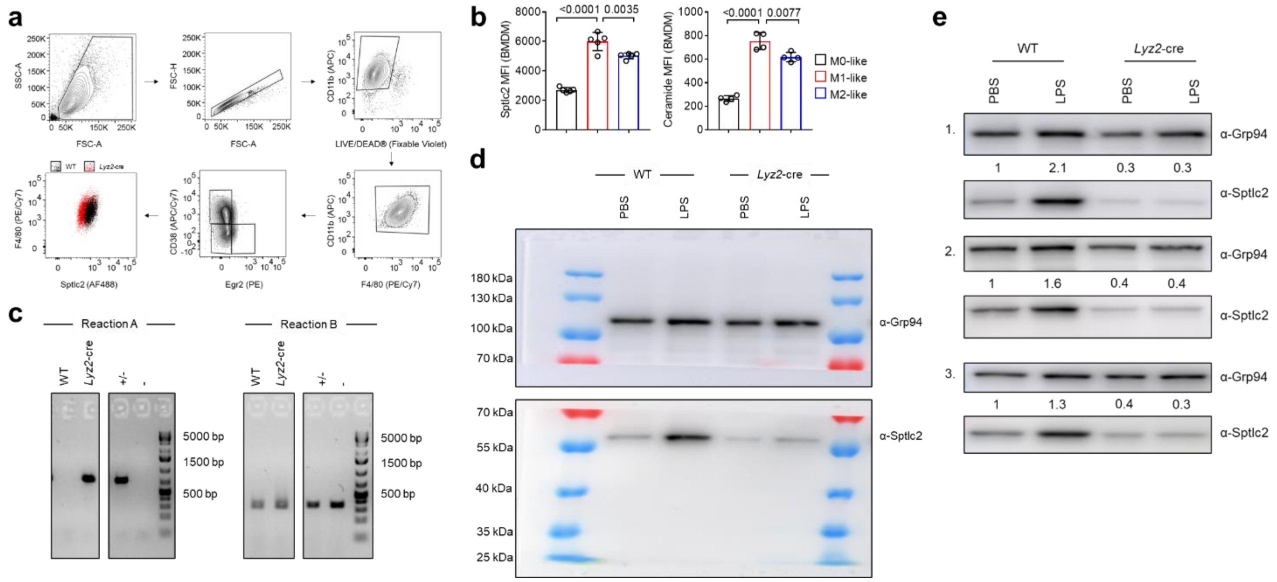

Supplementary Fig. 1, related to Figure 1 and Figure 3. Sptlc2 levels are diminished in BMDM of *Lyz2*-cre mice.

a Dot plots, showing the gating strategy used in Figure 1e, 3a, 3c and 3i to identify M0-like (live CD11b+F4/80+CD38-Egr2- cells), M1-like (live CD11b+F4/80+CD38+Egr2- cells) and M2-like (live CD11b+F4/80+CD38-Egr2+ cells) BMDM. Example showing M1-like macrophages. Last dot plot illustrates Sptlc2 staining in M1-like WT or *Lyz2*-cre BMDM in black or red, respectively.

b More replicates for flow cytometry results shown in Figure 1e (Sptlc2: n=5 biological replicates; Ceramide n=4 biological replicates).

c Representative genotype analysis of the WT or *Lyz2*-cre mice in Figure 1f. Primers were used according to the protocol for B6.129P2-Lyz2tm1(cre)Ifo/J from Jackson Laboratory (additional primer details for reactions A and B can be found in the manufacturer's instructions). Heterozygous ~700 bp and ~350 bp; wildtype ~350 bp; +/-, positive control (heterozygous); -, negative control (wildtype).

d Uncropped immunoblots shown in Figure 1f.

e More replicates for immunoblots shown in Figure 1f (n=3 biological replicates). The data are presented as mean ± SD (b). Statistical comparisons were performed with one-way ANOVA tests

(b; for simultaneous comparisons of more than two groups). Source data are provided as a Source Data file.

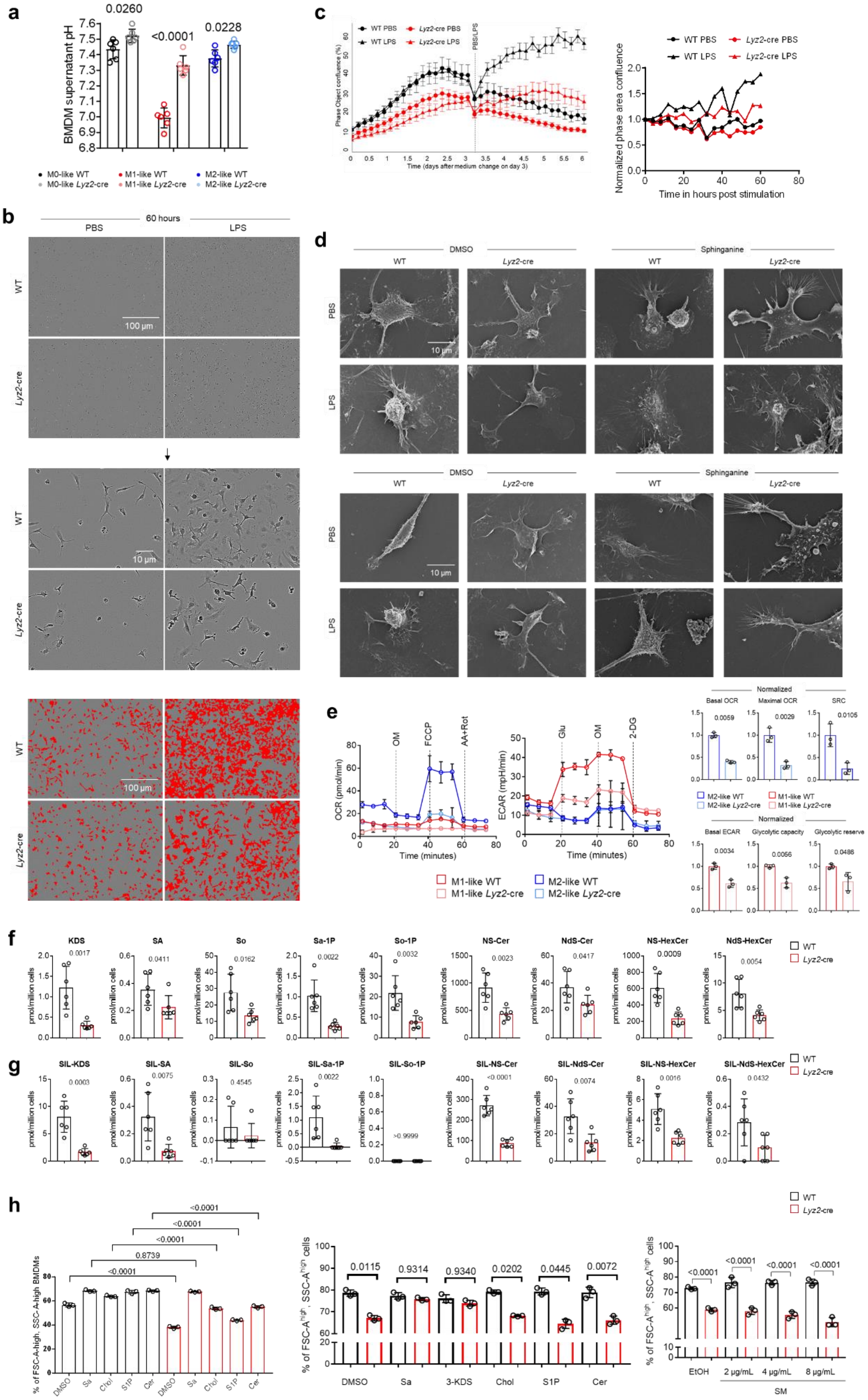
a
0.0260
<0.0001
0.0228
BMDM supernatant pH
M0-like WT
M0-like Lyz2-cre
M1-like WT
M1-like Lyz2-cre
M2-like WT
M2-like Lyz2-cre
b
60 hours
PBS
LPS
WT
Lyz2-cre
100 µm
10 µm
c
WT PBS
WT LPS
Lyz2-cre PBS
Lyz2-cre LPS
PBS/LPS
Phase Object confluence (%)
Time (days after medium change on day 3)
Normalized phase area confluence
Time in hours post stimulation
d
DMSO
Sphinganine
WT
Lyz2-cre
PBS
LPS
10 µm
e
OM
FCCP
AA+Rot
OCR (pmol/min)
Time (minutes)
Glu
2-DG
ECAR (mpH/min)
Normalized
Basal OCR
Maximal OCR
SRC
0.0059
0.0029
0.0105
M2-like WT
M2-like Lyz2-cre
M1-like WT
M1-like Lyz2-cre
Basal ECAR
Glycolytic capacity
Glycolytic reserve
0.0034
0.0056
0.0486
f
KDS
SA
So
Sa-1P
So-1P
NS-Cer
NdS-Cer
NS-HexCer
NdS-HexCer
0.0017
0.0411
0.0162
0.0022
0.0032
0.0023
0.0417
0.0009
0.0054
pmol/million cells
WT
Lyz2-cre
g
SIL-KDS
SIL-SA
SIL-So
SIL-Sa-1P
SIL-So-1P
SIL-NS-Cer
SIL-NdS-Cer
SIL-NS-HexCer
SIL-NdS-HexCer
0.0003
0.0075
0.4545
0.0022
>0.9999
<0.0001
0.0074
0.0016
0.0432
h
<0.0001
<0.0001
<0.0001
0.8739
<0.0001
% of FSC-A-high, SSC-A-high BMDMs
DMSO
Sa
Chol
S1P
Cer
0.0115
0.9314
0.9340
0.0202
0.0445
0.0072
% of FSC-A^{high}, SSC-A^{high} cells
3-KDS
EtOH
2 µg/mL
4 µg/mL
8 µg/mL
SM

Supplementary Fig. 2, related to Figure 2. ***Sptlc2*** **deficiency impairs M1-like BMDM growth.**

a More replicates for results shown in Figure 2a (n=6 biological replicates).

b Representative Incucyte images of WT or *Lyz2*-cre BMDM 60 hours after PBS or LPS treatment, without and with the confluency mask (red) overlaid, as shown in Figure 2b and enlarged images without the confluency mask.

c Line graph (left), showing non-normalized confluency of PBS and LPS-treated WT or *Lyz2*-cre BMDM over time for 6 days (starting after a medium change on day 3). The medium was changed after another 3 days, when PBS or LPS was added (indicated by the dashed line; see decrease in confluency). In Figure 2b, confluency was normalized to this time point. Line graph (right), showing more replicates for results shown in Figure 2b (normalized; n=4 biological replicates).

d More replicates for results shown in Figure 2c.

e More replicates for results shown in Figure 2d (n=3 biological replicates).

f-g Bar graphs, showing levels of endogenous (f) and stable isotope labeled (g) sphingolipids after LPS stimulation (n=6 biological replicates). Bar graphs show same data as the heat map in Figure 2f and include statistics.

h More replicates for results shown in Figure 2g. Bar graphs, showing percentages of FSC-A^{high}, SSC-A^{high} WT or *Lyz2*-cre LPS-activated BMDM after DMSO, sphinganine (Sa), cholesterol (Chol), sphingosine-1-phosphate (S1P), ceramide (Cer) or sphingomyelin (SM) supplementation for 3 days (n=3 biological replicates). The data are presented as mean ± SD (a, c, e-h) and pooled from 2 independent experiments (c, f-h). Statistical comparisons were performed with one-way ANOVA tests (a, h; for simultaneous comparisons of more than two groups), two-tailed unpaired Student's *t*-tests (e, f: all except Sa, Sa-1P; g: SIL-Sa, SIL-Sa-1P, SIL-So-1P; data points were normally distributed) and two-tailed Mann-Whitney U tests (f: Sa, Sa-1P; g: SIL-Sa, SIL-Sa-1P, SIL-So-1P; data points were not normally distributed). Source data are provided as a source Data file.

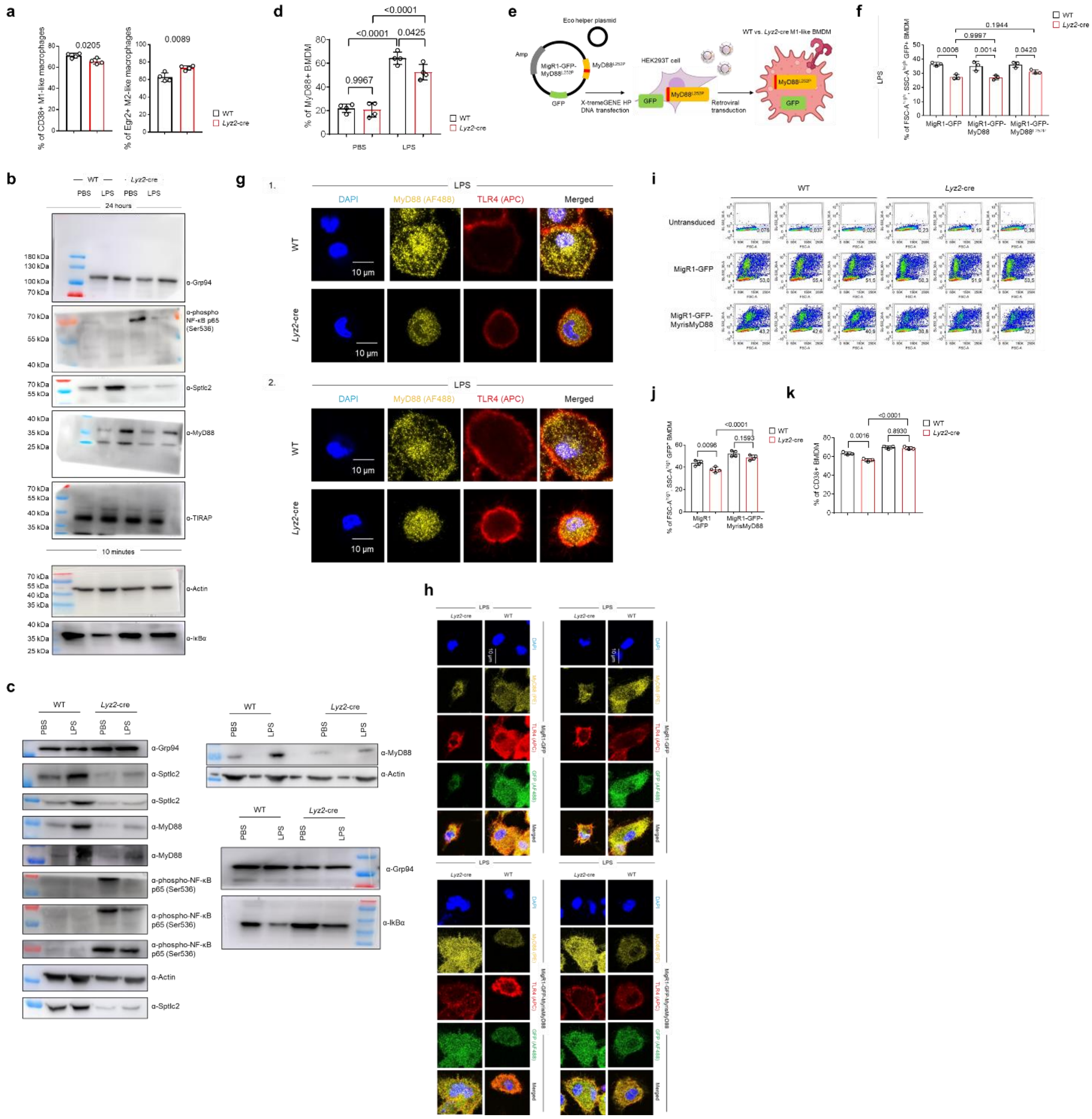

Supplementary Fig. 3, related to Figure 3. *Sptlc2* deficiency inhibits M1-like macrophage growth by preventing MyD88 recruitment to TLR4, which cannot be entirely rescued through overexpression of WT or L252P-mutated MyD88.

a More replicates for results shown in Figure 3a (n=4 biological replicates).

b Uncropped immunoblots shown in Figure 3b.

c More replicates for results shown in Figure 3b.

d More replicates for results shown in Figure 3c (n=4 biological replicates).

e Illustration of the experimental design in Supplementary Fig. 3f. WT or L252P-mutated MyD88 were overexpressed in WT or *Lyz2*-cre BMDM with X-treme GENE^{TM} HP transfection of HEK293T cells with MigR1-GFP-MyrisMyD88 and the eco helper plasmid, and subsequent retroviral transduction with viral particles of WT or *Lyz2*-cre BMDM.

f Bar graph, showing percentages of FSC-A^{high}, SSC-A^{high} GFP+ LPS-stimulated BMDM after overexpression of MigR1-GFP, MigR1-GFP-MyD88 or MigR1-GFP-MyD88^{L252P} (n=3 biological replicates).

g More replicates for results shown in Figure 3d (n=2 biological replicates).

h More replicates for results shown in Figure 3f (n=2 biological replicates).

i More replicates for results shown in Figure 3g (n=3 biological replicates).

j More replicates for results shown in Figure 3h (n=4 biological replicates).

k More replicates for results shown in Figure 3i (n=3 biological replicates). Data are from 2 (g, h) or 3 (c, f) independent experiments and presented as mean ± SD (a, d, f, j, k). Statistical comparisons were performed with two-tailed unpaired Student's *t*-tests (a; data points were normally distributed) and one-way ANOVA tests (d, f, j, k; for simultaneous comparisons of more than two groups). Supplementary figure 3e created with BioRender.com released under a Creative Commons Attribution-NonCommercial-NoDerivs 4.0 International license. Source data are provided as a Source Data file.

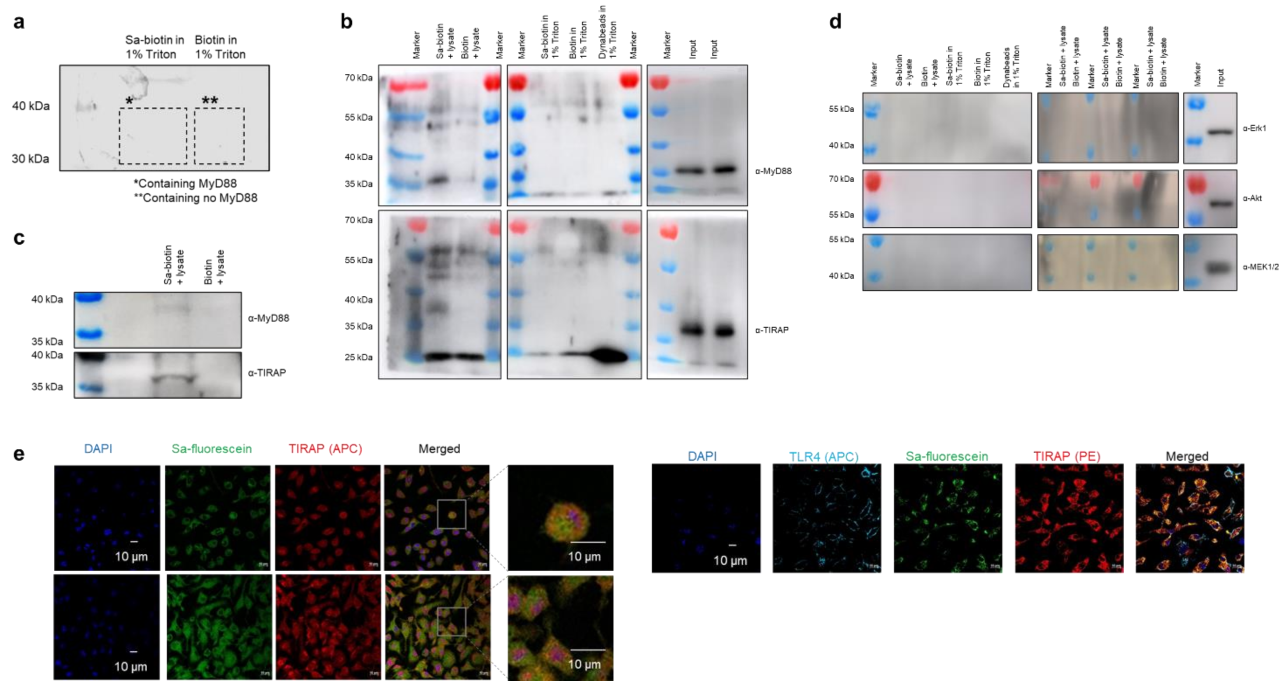

Supplementary Fig. 4, related to Figure 4. Sphinganine physically interacts with TIRAP and MyD88.

a More replicates for results shown in Figure 4b (n=1).

b Uncropped immunoblots shown in Figure 4c.

c More replicates for results shown in Figure 4c.

d Immunoblot analysis of stripped membranes stained for Erk1, Akt and MEK1/2 after sphinganine (Sa)-biotin and biotin control pulldown to ensure the specificity of the pulldown (n=5 biological replicates).

e More replicates for results shown in Figure 4d (n=3 biological replicates). The results are pooled from 2 independent experiments (d-e). Source data are provided as a Source Data file.

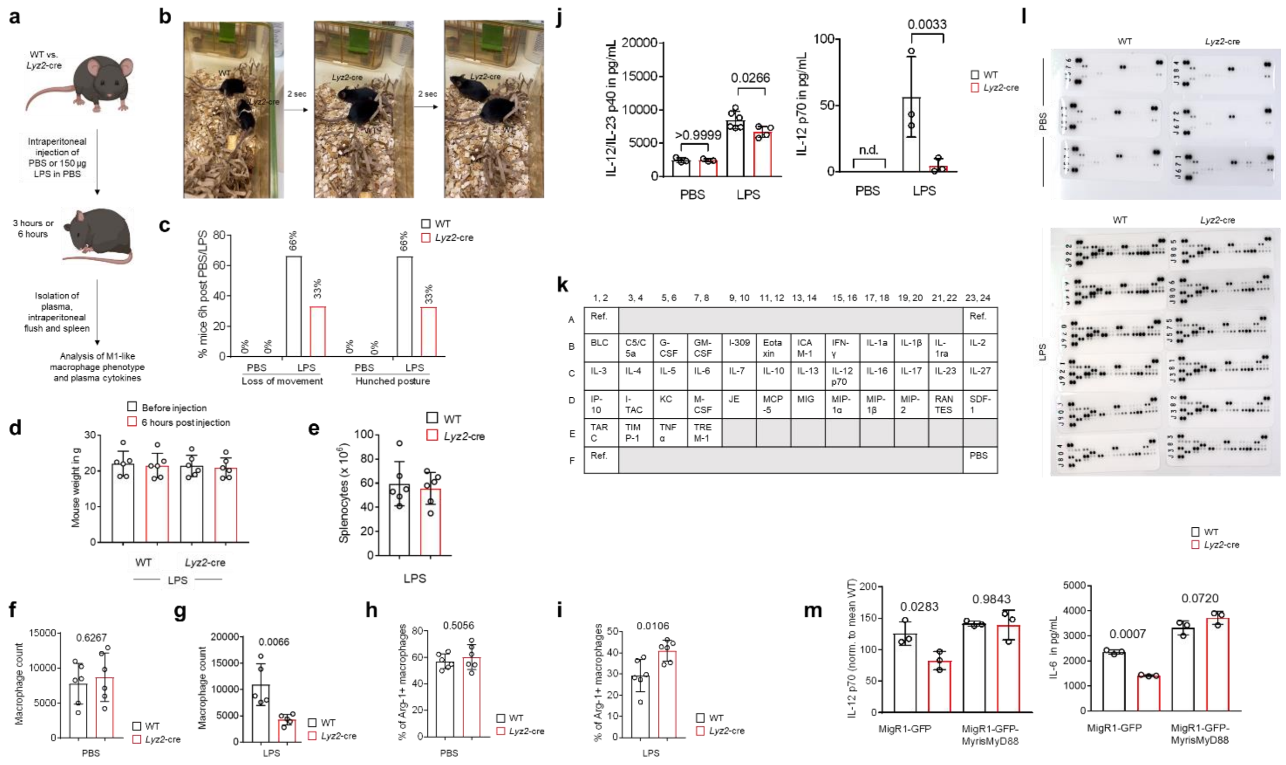

Supplementary Fig. 5, related to Figure 1 and Figure 5. *Sptlc2*-deficient mice, showing attenuated LPS-induced sepsis symptoms and plasma cytokine levels.

a Illustration of experimental design in Figure 1e, 5b-h and Supplementary Fig. 5b-m. LPS was intraperitoneally injected into WT or *Lyz2*-cre mice, and the movement and posture of the mice were monitored.

b More replicates for results shown in Figure 5b.

c More replicates for results shown in Figure 5c (PBS: n=3 biological replicates, LPS: n=6 biological replicates).

d Bar graphs, showing the weight of mice before and 6 hours after LPS injection (n=6 biological replicates).

e Bar graphs, showing splenocyte counts in millions 6 hours after LPS injection (n=6 biological replicates).

f Bar graph, showing relative WT or *Lyz2*-cre intraperitoneal macrophage counts 6 hours after PBS injection, measured by flow cytometry (n=6 biological replicates).

g More replicates for results shown in Figure 5e (n=5 biological replicates).

h Bar graph, showing percentages of Arg-1+ macrophages collected by intraperitoneal flushing 6 hours after PBS injection (n=6 biological replicates).

i More replicates for results shown in Figure 5f (n=6 biological replicates).

j More replicates for results shown in Figure 5g (PBS: n=3 biological replicates (IL-12 p70 below detection limit); LPS WT IL-12/23 p40: n=6 biological replicates; LPS *Lyz2*-cre IL-12/23 p40: n=4 biological replicates; LPS IL-12 p70: n= 3 biological replicates).

k Overview depicting the layout of the cytokine array shown in Figure 5h, Supplementary Fig. 5l and 6d.

l Uncropped cytokine array membranes of WT or *Lyz2*-cre mice after PBS or LPS injection. Membranes of LPS-injected mice were quantified with FIJI in Figure 5h.

m More replicates for results shown in Figure 5i (n=3 biological replicates). The data are presented as mean ± SD (d-j, m) and pooled from 2 independent experiments (d-f, h, l). Statistical comparisons were performed with unpaired two-tailed Student's *t*-tests (f-i; data points were normally distributed) or with one-way ANOVA tests (j, m; for simultaneous comparisons of more than two groups). Supplementary figure 5a created with BioRender.com released under a Creative Commons Attribution-NonCommercial-NoDerivs 4.0 International license. Source data are provided as a Source Data file.

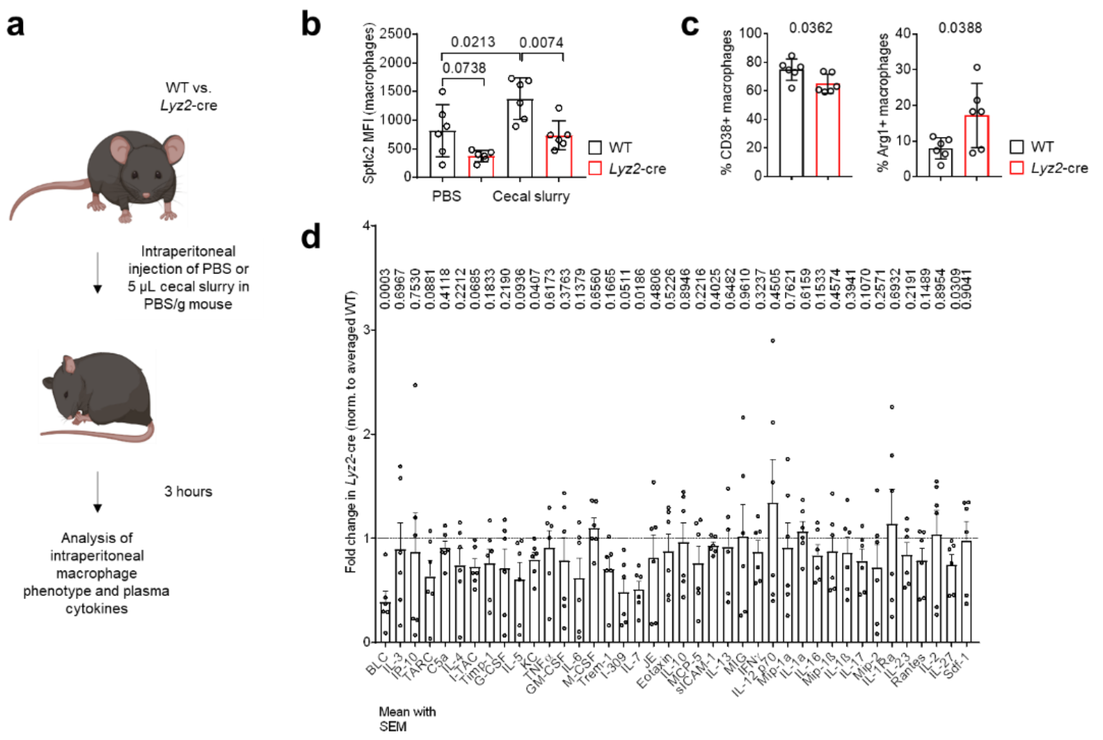

Supplementary Fig. 6 Deficiency in *Sptlc2* alleviates pro-inflammatory M1-like macrophage phenotype and cytokine production in cecal slurry-induced polymicrobial sepsis

a Experimental design of the polymicrobial sepsis model. Cecal slurry was intraperitoneally injected into WT or *Lyz2*-cre mice. Mice were sacrificed 3 hours post injection, and samples for cytokine array and flow cytometry analyses were isolated.

b Bar graph, showing MFI of Sptlc2 in WT or *Lyz2*-cre intraperitoneal macrophages 3 hours post injection, measured by flow cytometry (n=6 biological replicates).

c Bar graph, showing percentages of CD38+ or Arg-1+ macrophages collected by intraperitoneal flushing 6 hours after cecal slurry injection (n=6 biological replicates).

d Bar graph, showing the pixel density of cytokine dots in *Lyz2*-cre plasma normalized to WT plasma. A black dashed line separates up- and down-regulated cytokines in *Lyz2*-cre mice. Cytokine array blots spotted in duplicate with capture antibodies to specific target proteins, probed with plasma from WT or *Lyz2*-cre mice 3 hours after cecal slurry injection (n=6 biological replicates). The layout of the array can be found in Supplementary Fig. 5k. The data are presented

as mean ± SD (b-c) or mean + SEM (d). The results are pooled from 2 independent experiments with 3 pairs of mice in each experiment (b-d). Statistical comparisons were performed with one-way ANOVA tests (b; for simultaneous comparisons of more than two groups) and unpaired two-tailed Student's *t*-tests (c-d; data points were normally distributed). Supplementary figure 6a created with BioRender.com released under a Creative Commons Attribution-NonCommercial-NoDerivs 4.0 International license. Source data are provided as a Source Data file.

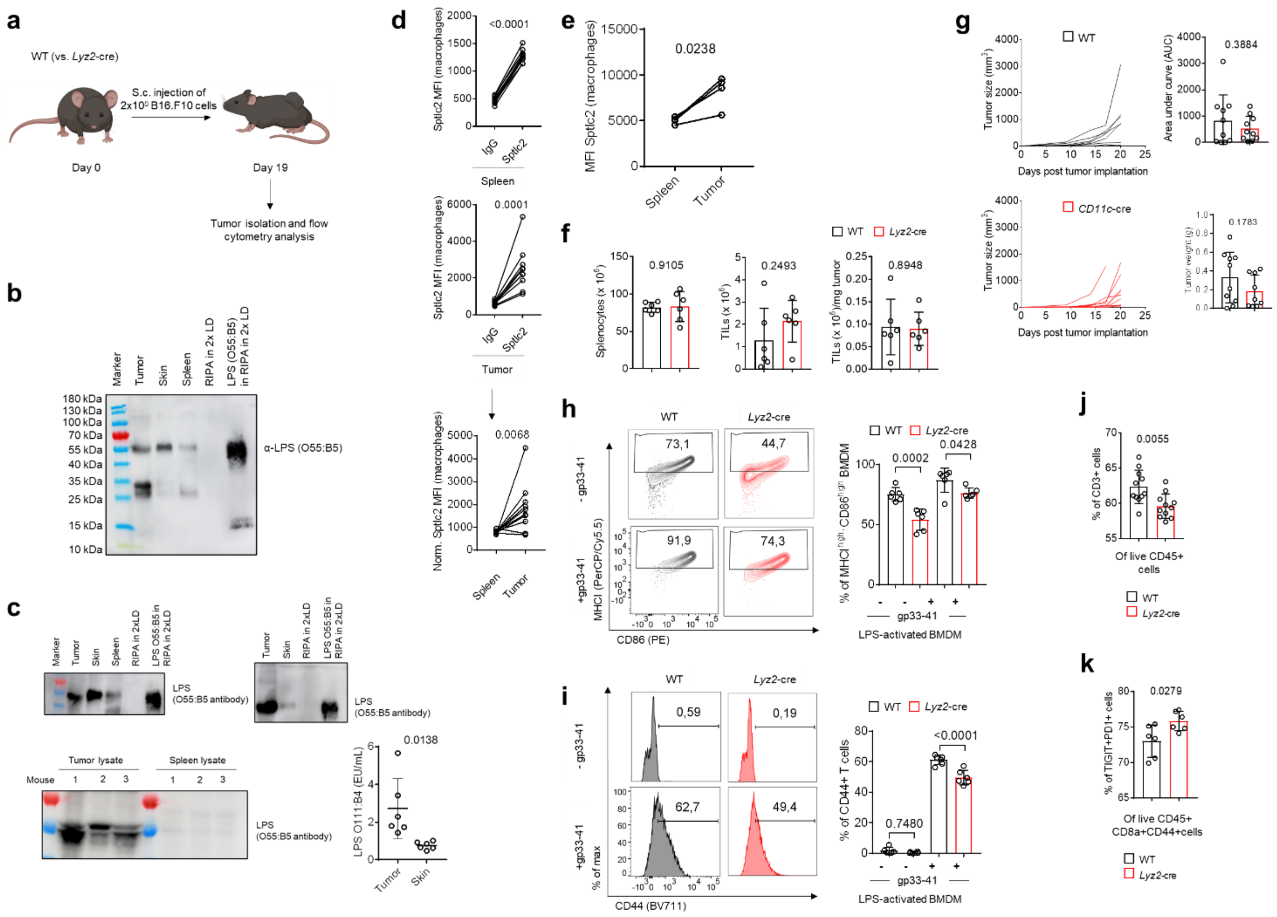

Supplementary Fig. 7, related to Figure 6. Myeloid cell-specific Sptlc2 shapes the anti-tumor immune response in the B16-F10 melanoma mouse model.

a Experimental design in Figure 6.

b Uncropped immunoblots shown in Figure 6a.

c More replicates for results shown in Figure 6a (Immunoblots n=5; LAL assay n=6 biological replicates).

d Graphs, showing Sptlc2 MFI of macrophages from the spleen and tumors, as shown in Figure 6b, including isotype control (n=10 biological replicates).

e More replicates for results shown in Figure 6b (n=4 biological replicates).

f Bar graphs, showing counts of splenocytes, tumor-infiltrating leukocytes (TILs; live CD45+ cells in tumors) and TILs/mg tumor of WT or *Lyz2*-cre mice from the B16-F10 tumor model (n=6 biological replicates).

g Line graphs, showing B16-F10 melanoma growth in WT or *CD11c*-cre mice. Bar graphs, showing the area under the curve (AUC) of the tumor growth plots and the tumor weight at the end-point on day 20 (n=10; weight (*CD11c*-cre): n=9 biological replicates).

h Histograms and bar graphs, showing percentages of MHCI^{high}, CD86^{high} WT or *Lyz2*-cre BMDM from co-culture of LPS-activated, LCMV gp33-41-pulsed BMDM and P14 CD8+ T cells (n=6 biological replicates).

i Histograms and bar graphs, showing percentages of CD44+ CD8+ T cells from co-culture of LPS-activated, LCMV gp33-41-pulsed BMDM and P14 CD8+ T cells (n=6 biological replicates).

j-**k** Bar graphs, showing percentages of CD3+ T cells among all live CD45+ WT or *Lyz2*-cre cells (n=11 biological replicates; j) and percentages of TIGIT+PD-1+ cells among all live CD45+CD8+CD44+ WT or *Lyz2*-cre cells (n=6 biological replicates; k) from the B16 tumors. Data are presented as symbols with connecting lines highlighting paired samples (d-e) and mean ± SD (c, f-k). Data are pooled from 2 (f-k) or 3 (immunoblots in c) independent experiments. Statistical comparisons were performed with two-tailed Student's *t*-tests (paired in d-e; unpaired in c, f, g, j-k; data points were normally distributed) and one-way ANOVA tests (h-i; for simultaneous comparisons of more than two groups). Supplementary figure 7a created with BioRender.com released under a Creative Commons Attribution-NonCommercial-NoDerivs 4.0 International license. Source data are provided as a Source Data file.
