# Supplementary material for: Sphinganine recruits TLR4 adaptors in macrophages and promotes inflammation in murine models of sepsis and melanoma
Source: Nat Commun. 2024 Jul 18;15:6067. doi: 10.1038/s41467-024-50341-w (PMC11258287; doi:10.1038/s41467-024-50341-w)
Supplement: Supplementary file 3 — Description of Additional Supplementary Files [file 41467_2024_50341_MOESM3_ESM.pdf]

**Description of Additional Supplementary Files**

**File Name: Supplementary Data 1**
**Description:** List of metabolites identified in M0-like, M1-like and M2-like WT BMDM (24h of stimulation; N=6). Related to Figure 1b.

**File Name: Supplementary Data 2**
**Description:** List of proteins identified after Sa-biotin IP in M1-like WT BMDM. Related to Figure 4b.

**File Name: Supplementary Movie 1**
**Description:** Video of LPS-induced sepsis mouse model

**File Name: Supplementary Movie 2**
**Description:** Video of more replicates from LPS-induced sepsis mouse model
