# Supplementary material for: Sphinganine recruits TLR4 adaptors in macrophages and promotes inflammation in murine models of sepsis and melanoma
Source: Nat Commun. 2024 Jul 18;15:6067. doi: 10.1038/s41467-024-50341-w (PMC11258287; doi:10.1038/s41467-024-50341-w)
Supplement: Supplementary file 8 — Reporting Summary [file 41467_2024_50341_MOESM8_ESM.pdf]

nature portfolio

Corresponding author(s): Dr. Guoliang Cui
Dr. Marvin Hering

Last updated by author(s): Jun 14, 2024

# Reporting Summary

Nature Portfolio wishes to improve the reproducibility of the work that we publish. This form provides structure for consistency and transparency in reporting. For further information on Nature Portfolio policies, see our Editorial Policies and the Editorial Policy Checklist.

## Statistics

For all statistical analyses, confirm that the following items are present in the figure legend, table legend, main text, or Methods section.

| n/a | Confirmed | |
|---|---|---|
| ☐ | ☒ | The exact sample size (*n*) for each experimental group/condition, given as a discrete number and unit of measurement |
| ☐ | ☒ | A statement on whether measurements were taken from distinct samples or whether the same sample was measured repeatedly |
| ☐ | ☒ | The statistical test(s) used AND whether they are one- or two-sided<br>*Only common tests should be described solely by name; describe more complex techniques in the Methods section.* |
| ☒ | ☐ | A description of all covariates tested |
| ☐ | ☒ | A description of any assumptions or corrections, such as tests of normality and adjustment for multiple comparisons |
| ☐ | ☒ | A full description of the statistical parameters including central tendency (e.g. means) or other basic estimates (e.g. regression coefficient) AND variation (e.g. standard deviation) or associated estimates of uncertainty (e.g. confidence intervals) |
| ☒ | ☐ | For null hypothesis testing, the test statistic (e.g. *F*, *t*, *r*) with confidence intervals, effect sizes, degrees of freedom and *P* value noted<br>*Give P values as exact values whenever suitable.* |
| ☒ | ☐ | For Bayesian analysis, information on the choice of priors and Markov chain Monte Carlo settings |
| ☒ | ☐ | For hierarchical and complex designs, identification of the appropriate level for tests and full reporting of outcomes |
| ☒ | ☐ | Estimates of effect sizes (e.g. Cohen's *d*, Pearson's *r*), indicating how they were calculated |

*Our web collection on statistics for biologists contains articles on many of the points above.*

## Software and code

Policy information about availability of computer code

| | |
|---|---|
| Data collection | We used FACS Diva Software (version 9, BD Biosciences) to collect FACS data.<br>Western blot data were collected by Fusion (FX6 Edge, Vilber).<br>Microscopy data were collect in ZEN Blue Zeiss software. |
| Data analysis | Raw RNA sequencing counts were downloaded from Gene Expression Omnibus (GEO) using the accession code GSE140610. Normalization and differential gene expression analysis were performed using the edgeR package version 3.28.1. To filter out lowly expressed genes, a counts-per-million (cpm) threshold was set to 1 before normalization. Normalized RNA sequencing counts were downloaded from GEO using the accession code GSE139913. FACS data were analyzed using Flowjo software (version 10.lrl). Western blot data were analyzed using Fusion FX6 Edge. Microscopy data were anylzed in ZEN Blue and FIJI/ImageJ software. |

For manuscripts utilizing custom algorithms or software that are central to the research but not yet described in published literature, software must be made available to editors and reviewers. We strongly encourage code deposition in a community repository (e.g. GitHub). See the Nature Portfolio guidelines for submitting code & software for further information.

## Data

Policy information about availability of data

All manuscripts must include a data availability statement. This statement should provide the following information, where applicable:

- Accession codes, unique identifiers, or web links for publicly available datasets
- A description of any restrictions on data availability
- For clinical datasets or third party data, please ensure that the statement adheres to our policy

The previously published sequencing data are available online at Gene Expression Omnibus (GSE139913 and GSE140610).

## Research involving human participants, their data, or biological material

Policy information about studies with human participants or human data. See also policy information about sex, gender (identity/presentation), and sexual orientation and race, ethnicity and racism.

| | |
|---|---|
| Reporting on sex and gender | no human participants |
| Reporting on race, ethnicity, or other socially relevant groupings | no human participants |
| Population characteristics | no human participants |
| Recruitment | no human participants |
| Ethics oversight | no human participants |

Note that full information on the approval of the study protocol must also be provided in the manuscript.

# Field-specific reporting

Please select the one below that is the best fit for your research. If you are not sure, read the appropriate sections before making your selection.

☒ Life sciences ☐ Behavioural & social sciences ☐ Ecological, evolutionary & environmental sciences

For a reference copy of the document with all sections, see nature.com/documents/nr-reporting-summary-flat.pdf

# Life sciences study design

All studies must disclose on these points even when the disclosure is negative.

| | |
|---|---|
| Sample size | Per experiment sample sizes were determined to be big enough to give statistically valid results. Results were always confirmed in repeats of the experiments with same sample sizes. No formal statistical methods were used to predetermine sample sizes but our sample sizes are similar to those reported in previous publications. |
| Data exclusions | We did not exclude data. |
| Replication | Per experiment with the same settings all experiments resulted in the same results. Experiments were repeated twice or three times, as indicated in the figure legends. |
| Randomization | Allocoation was random in that wt or ko mice were randomly selected to be injected with e.g. LPS, cecal slurry or tumor cells or controls<br>For the comparison between wildtype and knockout mice, littermate mice were allocated into 2 groups based on genotypes (namely wildtype and knockout mice). We did not use a randomization protocol for the rest of the experiments. |
| Blinding | Data collection and analysis were not performed blind to the conditions of the experiments, because investigators who planned the experiments also performed them. |

# Behavioural & social sciences study design

All studies must disclose on these points even when the disclosure is negative.

| | |
|---|---|
| Study description | No behavioural & social studies are described in this study |
| Research sample | No behavioural & social studies are described in this study |

| | |
|---|---|
| Sampling strategy | No behavioural & social studies are described in this study |
| Data collection | No behavioural & social studies are described in this study |
| Timing | No behavioural & social studies are described in this study |
| Data exclusions | No behavioural & social studies are described in this study |
| Non-participation | No behavioural & social studies are described in this study |
| Randomization | No behavioural & social studies are described in this study |

# Ecological, evolutionary & environmental sciences study design

All studies must disclose on these points even when the disclosure is negative.

| | |
|---|---|
| Study description | No Ecological, evolutionary & environmental sciences are described in this study |
| Research sample | No Ecological, evolutionary & environmental sciences are described in this study |
| Sampling strategy | No Ecological, evolutionary & environmental sciences are described in this study |
| Data collection | No Ecological, evolutionary & environmental sciences are described in this study |
| Timing and spatial scale | No Ecological, evolutionary & environmental sciences are described in this study |
| Data exclusions | No Ecological, evolutionary & environmental sciences are described in this study |
| Reproducibility | No Ecological, evolutionary & environmental sciences are described in this study |
| Randomization | No Ecological, evolutionary & environmental sciences are described in this study |
| Blinding | No Ecological, evolutionary & environmental sciences are described in this study |

Did the study involve field work? ☐ Yes ☒ No

## Field work, collection and transport

| | |
|---|---|
| Field conditions | n/a |
| Location | n/a |
| Access & import/export | n/a |
| Disturbance | n/a |

# Reporting for specific materials, systems and methods

We require information from authors about some types of materials, experimental systems and methods used in many studies. Here, indicate whether each material, system or method listed is relevant to your study. If you are not sure if a list item applies to your research, read the appropriate section before selecting a response.

## Materials & experimental systems

| n/a | Involved in the study | |
|---|---|---|
| ☐ | ☒ | Antibodies |
| ☐ | ☒ | Eukaryotic cell lines |
| ☒ | ☐ | Palaeontology and archaeology |
| ☐ | ☒ | Animals and other organisms |
| ☒ | ☐ | Clinical data |
| ☒ | ☐ | Dual use research of concern |
| ☒ | ☐ | Plants |

## Methods

| n/a | Involved in the study | |
|---|---|---|
| ☒ | ☐ | ChIP-seq |
| ☐ | ☒ | Flow cytometry |
| ☒ | ☐ | MRI-based neuroimaging |

# Antibodies

Antibodies used

CD11b (BioLegend, #101212 and #101226, M1/70; 1:400), F4/80 (Miltenyi Biotech, #130-116-547, #130-118-327 and BioLegend, #123114, BM8 and REA126; 1:400), CD38 (BioLegend, #102728 and Miltenyi Biotech, #130-128-224, REA616 and 90; 1:400), Egr2 (Miltenyi Biotech, #130-114-256, REA869; 1:100), CD45 (BioLegend, #103147, 30-F11; 1:400), B220 (Miltenyi Biotech, #130-110-711, REA755; 1:400), CD3 (Miltenyi Biotech, #130-116-530, REA641; 1:400), CD4 (BioLegend, #100406; 1:400), NK1.1 (Miltenyi Biotech, #130-116-533, PK136; 1:400), Ly6G (Miltenyi Biotech, #130-119-902, REA526), Ly6C (BioLegend, #128006, HK1.4; 1:200), IL-12 (Miltenyi Biotech, #130-102-163, REA136; 1:200), Ki-67 (BioLegend, #652424, 16A8; 1:400), MHCI (BioLegend, #111518, KH95; 1:400), MHCII (BioLegend, #107626 and #107608, M5/114.15.2; 1:400), CD45 (BioLegend, #103149, 30-F11; 1:400), CD44 (BioLegend, #103057, IM7; 1:200), TIGIT (BioLegend, #142104, 1G9; 1:100), CD8a (BioLegend, #100804, 5H10-1; 1:400), CD86 (BioLegend, #105008, GL-1; 1:400), Granzyme B (BioLegend, #372210, QA16A02; 1:400), IgG (BioLegend, #406421; 1:400), TNFα (BioLegend, #506306; 1:200), PD-1 (BioLegend, #135216; 1:400) and CD16/32 (BioLegend, #101330, 93; 1:500), Sptlc2 (rb; polyclonal; 1:400), ceramide (Enzo Life sciences, #ALX-804-196-T050, ms; MID 15B4; 1:100), MyD88 (rb; #D80F5; 1:100), Arg-1 (Cell Signaling Technology, #93668, rb; D4E3M; 1:100), anti-rb-AF647 (BioLegend, #406414; 1:200), -AF488 (BioLegend, #406416; 1:200) or -PE (BioLegend, #406421, all poly4064; 1:200), anti-mouse-BV421™ (#406517, RMM-1; 1:200), anti-Sptlc2 (Origene, #TA319780), anti-Grp94 (Cell Signaling Technology, #20292, D6X2Q), anti-Bcl-2 (Cell Signaling Technology, #3498, D17C4), anti-phospho-NF-κB p65(Ser536; Cell Signaling Technology, #3033, 93H1), anti-MyD88 (Cell Signaling Technology, #4283, D80F5), anti-TIRAP (Cell Signaling Technology, #13077, D6M9Z), anti-IκBα (Cell Signaling Technology, #4812, 44D4), anti-LPS (Thermo Fisher Scientific, #MA5-41631, C6), anti-Erk1 (Cell Signaling Technology, #4372), anti-Akt (Cell Signaling Technology, #9272), anti-MEK1/2 (Cell Signaling Technology, #9122), TIRAP (rb; CST, #D6M9Z) and MyD88 (rb; CST, #D80F5), anti-rb, poly4064 (anti-mouse, RMM-1), TLR4-APC (Thermo Fisher Scientific, #MTS510)

Validation

Antibodies used in this study were suggested by the manufacturer's for the species used (mouse) and validated in each experiment

# Eukaryotic cell lines

Policy information about cell lines and Sex and Gender in Research

Cell line source(s)

Prof. Dr. Hanspeter Pircher, Max Planck Institut für Immunbiologie und Epigenetik, Freiburg, Germany (B16-F10 melanoma cells)

Authentication

none were authenticated

Mycoplasma contamination

Cell lines not tested for mycoplasma contamination

Commonly misidentified lines
(See ICLAC register)

n/a

# Palaeontology and Archaeology

Specimen provenance

n/a

Specimen deposition

n/a

Dating methods

n/a

☐ Tick this box to confirm that the raw and calibrated dates are available in the paper or in Supplementary Information.

Ethics oversight

n/a

Note that full information on the approval of the study protocol must also be provided in the manuscript.

## Animals and other research organisms

Policy information about studies involving animals; ARRIVE guidelines recommended for reporting animal research, and Sex and Gender in Research

| | |
|---|---|
| Laboratory animals | This research complies with all relevant ethical regulations and all studies were performed in accordance with DKFZ regulations after approval by the German regional council at the Regierungspräsidium Karlsruhe (G266-19, G255-16, G236-21). Mice (mus musculus, C57BL/6) were maintained in the German cancer research center (DKFZ) specific-pathogen-free facility. The mice were housed in a 12 h on (7 am – 7 pm), 12 h off light cycle with room temperature of 20 °C – 24 °C and humidity of 45 – 65%. The Sptlc2Flox/Flox mice were kindly provided by Professor Xian-cheng Jiang (SUNY Downstate Medical Center, New York, USA) via Professor Vishwa Dixit (Yale University, New Haven, USA) and Professor Susan Kaech (Salk Institute for Biological Studies, La Jolla, USA). Exon 1 of Sptlc2 is flanked by two LoxP sites and is excised after crossing with a Cre-expression mouse strain. Lyz2-cre mice and CD11c-cre mice were from the Jackson Laboratory. For generation of myeloid cell- or dendritic cell-specific Sptlc2 knockout mice, Sptlc2Flox/Flox mice were bred with Lyz2-cre or CD11c-cre mice, respectively. Offspring from this breeding, which had one allele of Sptlc2 floxed, and which had a Lyz2- or CD11c-cre, were again bred with Sptlc2Flox/Flox mice to generate Sptlc2Flox/Flox Lyz2-cre or CD11c-cre mice. Wildtype littermate mice were used as controls. We used sex-matched and age-matched (7-17 week-old) mice for each individual experiment. In rare cases, mice with fighting wounds were excluded from the experimental analysis. The sample collection and processing were not performed in a blinded manner. |
| Wild animals | We did not use wild animals. |
| Reporting on sex | We used both male and female mice in this study. We used sex-matched mice for each individual experiment. |
| Field-collected samples | Study does not contain samples collected from the field. |
| Ethics oversight | All studies were performed in accordance with DKFZ regulations with approval by the German regional council at the Regierungspräsidium Karlsruhe. |

Note that full information on the approval of the study protocol must also be provided in the manuscript.

## Clinical data

Policy information about clinical studies

All manuscripts should comply with the ICMJEguidelines for publication of clinical research and a completedCONSORT checklist must be included with all submissions.

| | |
|---|---|
| Clinical trial registration | n/a |
| Study protocol | n/a |
| Data collection | n/a |
| Outcomes | n/a |

## Dual use research of concern

Policy information about dual use research of concern

### Hazards

Could the accidental, deliberate or reckless misuse of agents or technologies generated in the work, or the application of information presented in the manuscript, pose a threat to:

| No | Yes | |
|---|---|---|
| ☒ | ☐ | Public health |
| ☒ | ☐ | National security |
| ☒ | ☐ | Crops and/or livestock |
| ☒ | ☐ | Ecosystems |
| ☒ | ☐ | Any other significant area |

## Experiments of concern

Does the work involve any of these experiments of concern:

| No | Yes | |
|---|---|---|
| ☒ | ☐ | Demonstrate how to render a vaccine ineffective |
| ☒ | ☐ | Confer resistance to therapeutically useful antibiotics or antiviral agents |
| ☒ | ☐ | Enhance the virulence of a pathogen or render a nonpathogen virulent |
| ☒ | ☐ | Increase transmissibility of a pathogen |
| ☒ | ☐ | Alter the host range of a pathogen |
| ☒ | ☐ | Enable evasion of diagnostic/detection modalities |
| ☒ | ☐ | Enable the weaponization of a biological agent or toxin |
| ☒ | ☐ | Any other potentially harmful combination of experiments and agents |

# Plants

Seed stocks: n/a

Novel plant genotypes: n/a

Authentication: n/a

# ChIP-seq

## Data deposition

☐ Confirm that both raw and final processed data have been deposited in a public database such as GEO.

☐ Confirm that you have deposited or provided access to graph files (e.g. BED files) for the called peaks.

Data access links
*May remain private before publication.*: n/a

Files in database submission: n/a

Genome browser session (e.g. UCSC): n/a

## Methodology

Replicates: n/a

Sequencing depth: n/a

Antibodies: n/a

Peak calling parameters: n/a

Data quality: n/a

Software: n/a

# Flow Cytometry

## Plots

Confirm that:

- [x] The axis labels state the marker and fluorochrome used (e.g. CD4-FITC).
- [x] The axis scales are clearly visible. Include numbers along axes only for bottom left plot of group (a 'group' is an analysis of identical markers).
- [x] All plots are contour plots with outliers or pseudocolor plots.
- [x] A numerical value for number of cells or percentage (with statistics) is provided.

## Methodology

| | |
|---|---|
| Sample preparation | Single-cell suspensions were generated using cell strainers and syringes and Fc receptors were blocked for at least 15 minutes on ice with Ultra-LEAF™ Purified anti-mouse CD16/32 antibody in PBS supplemented with 1% FBS (FACS buffer) to prevent nonspecific antibody binding. For surface antigen staining, cells were incubated in FACS buffer with fluorophore-conjugated antibodies for at least 30 minutes on ice. Live/DEAD Fixable Dead Cell Stain kits were used to exclude the dead cells. When only surface antigens were stained, fixation buffer containing 4% paraformaldehyde was used. For staining of intracellular antigens, cells were fixed and permeabilized on ice for at least 30 minutes, and intracellular antigens were stained directly or via primary and secondary antibodies. Samples were washed and run on an LSR II or Canto flow cytometer and analyzed in FlowJo software (v10, FlowJo). |
| Instrument | Samples were washed and run on an LSR II or Canto flow cytometer. |
| Software | We used FACS Diva Software (version 9, BD Biosciences) to collect FACS data and analyzed data in FlowJo software (10.1r1). |
| Cell population abundance | No facsorting was performed in this study. |
| Gating strategy | Gates were first set based on FSC-A/SSC-A and doublets were excluded using FSC-H and FSC-A. Live/death dye was used to exclude dead cells. Myeloid cells were then gated for further analysis. More detailed gating strategies are shown as part of the main and supplementary figure set. All gating strategies are shown as illustrations in the figures. |

- [x] Tick this box to confirm that a figure exemplifying the gating strategy is provided in the Supplementary Information.

# Magnetic resonance imaging

## Experimental design

| | |
|---|---|
| Design type | n/a |
| Design specifications | n/a |
| Behavioral performance measures | n/a |

## Acquisition

| | |
|---|---|
| Imaging type(s) | n/a |
| Field strength | n/a |
| Sequence & imaging parameters | n/a |
| Area of acquisition | n/a |

Diffusion MRI ☐ Used ☒ Not used

## Preprocessing

| | |
|---|---|
| Preprocessing software | n/a |
| Normalization | n/a |
| Normalization template | n/a |
| Noise and artifact removal | n/a |
| Volume censoring | n/a |

## Statistical modeling & inference

Model type and settings: n/a

Effect(s) tested: n/a

Specify type of analysis: ☐ Whole brain ☐ ROI-based ☐ Both

Statistic type for inference: n/a

(See Eklund et al. 2016)

Correction: n/a

## Models & analysis

| n/a | Involved in the study |
|---|---|
| ☒ | ☐ Functional and/or effective connectivity |
| ☒ | ☐ Graph analysis |
| ☒ | ☐ Multivariate modeling or predictive analysis |

Functional and/or effective connectivity: n/a

Graph analysis: n/a

Multivariate modeling and predictive analysis: n/a
